# Science to the rescue or contingent progress? Comparing 10 years of public, expert and policy discourses on new and emerging science and technology in the United Kingdom

**DOI:** 10.1177/0963662517706452

**Published:** 2017-05-11

**Authors:** Melanie Smallman

**Affiliations:** University College London, UK

**Keywords:** governance, public dialogue, public discourse, public engagement

## Abstract

Over the past 10 years, numerous public debates on new and emerging science and technologies have taken place in the United Kingdom. In this article, we characterise the discourses emerging from these debates and compare them to the discourses in analogous expert scientific and policy reports. We find that while the public is broadly supportive of new scientific developments, they see the risks and social and ethical issues associated with them as unpredictable but inherent parts of the developments. In contrast, the scientific experts and policymakers see risks and social and ethical issues as manageable and quantifiable with more research and knowledge. We argue that these differences amount to two different sociotechnical imaginaries or views of science and how it shapes our world – an elite imaginary of ‘science to the rescue’ shared by scientists and policymakers and public counter-imaginary of ‘contingent progress’. We argue that these two imaginaries indicate that, but also help explain why, public dialogue has had limited impact on public policy.

## 1. Introduction

Over the past 20 years, as part of the United Kingdom’s attempts to move science in public from ‘deficit to dialogue’ (Smallman, 2014; Stilgoe et al., 2014), a series of ‘public dialogue’ events have taken place, where members of the public have been brought together to discuss new and emerging topics in science and technology. Most notably, since 2004, the UK government programme ‘Sciencewise’ has assembled ‘mini-publics’ and invited them to discuss, consider and express views on a particular aspect of new and emerging science or technology, with the aim of feeding these views into public policymaking. Topics have ranged from nanoscience and synthetic biology to energy use and data management, generating considerable debate and information about public perspectives on a full range of science issues.

While each of these public dialogue events has been studied and evaluated, these studies have tended to focus on the process of dialogue – whether particular groups have had a say, whether the discussions were framed by the participants and organisers (see, for example, Rowe, 2005; Rowe and Frewer, 2000) or focused on case studies or particular areas of science (Smallman, 2014). Many of these studies have concluded that evidence of ‘mini-public’ events influencing policy is rare (e.g. Goodin, 2006; Hansen and Allansdottir, 2011; Kurath and Gisler, 2009) or limited to endorsing a predetermined approach (Chilvers, 2012; Macnaghten and Chilvers, 2014; Stirling, 2007; Thorpe and Gregory, 2010; Wynne, 2006).

However, to fully evaluate the influence of such discussions on policy, understanding what is discussed in these public dialogue events – and how these discussions differs to those of more ‘expert’ sources of policy influence – would appear to be key, particularly if we are to understand any underlying mechanisms that exclude or include views. Furthermore, more than 10 years of public discussions on such a broad range of science topics also appears to offer insight into how people encounter and form views on new and emerging science and technologies. While there has been considerable interest in lay perspectives on science and technology, there has been much less work to characterise the corresponding ‘scientific’, ‘expert’ or ‘policy’ perspectives – arguably the views that public perspectives are contrasted against.

This article sets out to address these gaps. To begin, we look at the discourses within reports of public dialogue to ask how is science and technology discussed in these public dialogue events and what do these discussions tell us about how the public come to understand science. We then look at the way science and technology is discussed in the analogous scientific and policy documents, similarly identifying the key discourses. By comparing the three sets of discourses, we go on to ask what is similar and different in the perspectives of the public, scientific experts and policymakers and whether we can see any similarities, alignments or differences. In addition to characterising public, scientific and policy discussions about new and emerging technologies, this analysis will help us understand which views, if any, have been taken up by policy.

Beyond understanding how particular technologies are discussed, by taking a longer view across technologies, we ask whether the public, scientists and policymakers share the same understandings of the place of science in our society, the kind of future we are building with it and how it should be regulated. To do this, we use the concept of ‘Sociotechnical Imaginaries’, defined as follows:

*Collectively held, institutionally stabilised and publicly performed visions of desirable futures, animated by shared understandings of forms of social life and social order, attainable through and supportive of advances in science and technology. (Jasanoff, 2015)*

We argue that understanding the sociotechnical imaginaries underpinning each of the public, expert and policy discourses is important if we are to understand how public and scientists’ attitudes to science and technology differ, and whether and how public perspectives are reflected in policymaking.

## 2. Analysing public, scientific and policy discourses on new and emerging technologies

The UK national public dialogues on science and technology, most of which were sponsored by the government’s Sciencewise programme (www.sciencewise-erc.org.uk), have been well documented with each producing a report for policymakers. These reports (2002–2011) form Corpus A, the basis of our analysis of public discourses and sociotechnical imaginaries.

The public dialogue reports fed into or were accompanied by scientific ‘expert’ reports for policymakers, typically produced by learned societies. These documents formed Corpus B, the basis of analysis of ‘expert’ discourses and imaginaries.

The government responses to these two sets of reports formed Corpus C, upon which our examination of ‘policy’ discourses and sociotechnical imaginaries was based.

A full list of the document included is given in the Online Appendix. Topics include nanoscience, stem cells, geoengineering, synthetic biology, genetically modified (GM) crops and animal–human hybrid embryos.

It is important to note two points here: First, all three of these sources are ‘mediated’ and not verbatim transcripts of discussions. The reports are therefore likely to reflect only part of what has happened in the course of the discussions (Shaw et al., 2004) and, in particular, the parts of the discussion that the authors wish to display (Hilgartner, 2000). Nevertheless, documents are considered important sources for understanding the relationship between evidence and policy (D Evans, 2014; Freeman and Maybin, 2011) and in understanding sociotechnical imaginaries (Jasanoff, n.d.). Most importantly, informal interviews with policymakers indicate that written reports of public dialogues and expert deliberations are the main ways in which policymakers encounter these discussions.

Second, the categories of ‘public’, ‘expert’ and ‘policy’ are actor categories derived from the document authors rather than the analysts. ‘Public’ refers to the participants contributing to the public dialogue reports; ‘Expert’ includes natural and social scientists involved in the learned society reports; ‘Policy’ refers to the range of those involved in policymaking, including politicians and also government officials.

Given the size of the corpus, and to take an overarching but detailed view of the discussions in public dialogue, we used a computer-assisted text analysis (CATA) approach, using the software IRAMUTEQ (Ratinaud and Dejean, 2009; Ratinaud and Marchand, 2012). This approach is well established in political science research (e.g. Bara et al., 2007; Laver et al., 2003; Schonhardt-Bailey, 2005) and has previously been used in Public Understanding of Science research to identify media frames (Parales-Quenza, 2004), analyse answers to open-ended survey questions (Stoneman et al., 2013), understand long-term publishing trends (Smallman, 2014) and investigate researchers’ interpretation of impact (Terämä et al., 2016).

The theoretical model behind CATA is the Word Space Model (Chartier and Meunier, 2011) – a computational model based on structural linguistics (De Saussure, 1916) describing how words’ meanings are derived by looking at how words are distributed and situated across a large text. Since the meaning of a word is built through its use and words with similar co-occurrence patterns have similar meanings, the model argues that if different stakeholders have different meanings attached to particular words, then these differences will be reflected in the way they use these words. As such, it will be possible to identify discourses by looking at the way words group together.

To help in this process, IRAMUTEQ produces a statistical map of the corpus, based upon the pattern of words in that corpus, which the researcher then interprets and analyses (full technical details of the software methodology are given in Ratinaud and Dejean, 2009 and Ratinaud and Marchand, 2012).

First, the ‘content’ words (nouns, adjectives, verbs and adverbs) are separated from the ‘function’ words (articles, prepositions and pronouns) to form the basis of the analysis. The corpus is then broken into text segments of fixed length, mimicking sentences. The presence/absence of every word in the corpus in each sentence is tabulated, and this table is split into the two most dissimilar groups or ‘classes’ using descending hierarchical clustering (DHC). The biggest of these two classes is split again into another two classes and so forth, until 10 (default) rounds of splitting have been undergone or until no further splits can be made (a setting of 8, 10 and 12 rounds was tested with sample data, and 10 was not found to be a limiting factor).

The statistical map IRAMUTEQ produces includes the following: lists of the most significant words in the text, grouped into clusters (or classes) showing the words that most often appear in a sentence together; chi-squared measures showing the relationship between the words and the clusters; and sentences from the original text most associated with each class.

It is important to emphasise that IRAMUTEQ does not produce ‘automatic’ results. Instead, the researcher uses all of this material, along with the original text, to build an understanding of the texts and discourses and to identify abductively the most plausible inferences in the data. Following the protocol of Smallman (2014), we drew at least two possible interpretations for each class, tested them against the original text and amended and accepted the most plausible interpretations. We produced interpretative labels for each class, along with ‘illustrative statements’, constructed from the word lists of each class. The interpretive labels and the 10 words most closely associated with the classes are given below. Fuller details including the 50 words most associated with each of the classes are given in the online appendix.

The classes produced represent the most distinct discourses within the corpuses – we identified five classes or discourses within the public documents, five within the scientific expert documents and four within the policy documents.

Taking an overview of the discourses emerging from each corpus, we then identified the underlying sociotechnical imaginaries for each actor category (i.e. public, experts and policymakers), by asking questions about six key features of the discourses, which have been developed from Jasanoff and Kim’s (2009, 2015) descriptions of the key features of sociotechnical imaginaries:

1. What is the ontological focus of the discourses from this actor category?
2. How is progress described?
3. How are social and ethical issues viewed?
4. How are risk and uncertainty understood?
5. What is the role of industry?
6. What is the role of government?

## 3. Results

### A Public discourses

Analysing the 18 public dialogue reports produced five classes, reflecting the five most distinct discourses within the corpus (Table 1).

**Table 1.** Summary of analysis of public dialogue reports.

Class	Illustrative statement	Ten most significant words	Documents associated
Class A1 (15.1%): Drugs – Cure or cause, treating medical or social problems?	‘I know that drugs like heroin need to be illegal because of the harm they cause – especially for vulnerable young people. But I suppose we could say the same about other recreational drugs like alcohol or nicotine, which can also be addictive. And while prescription drugs can help people with mental health problems, where do we draw the line with social problems? Should people be allowed to take drugs that help them do well in education, for instance?’	drug young recreational outreach user person Belfast illicit child parent	Drugsfutures (2006)
Class A2 (25.5%): ‘Reaching potential whilst minimising risk’	‘These technologies show a lot of promise to develop medical treatments in the future. But there are also risks (some unknown) and the private companies involved will be driven by the need to make a profit. We need to think about how we govern and regulate this tension’.	application area treatment biology potential science synthetic disease fund nanotechnology	BBSRC Synthetic Biology (2009) Stem Cell Dialogue (2007) ScienceHorizons Small Groups (2006)
Class A3: Precautionary in Principle	‘We need to know a lot more about these new technologies and to discuss them further before we can make decisions and policy about them. We need independent advice about whether they will work and what the costs and side effects will be’.	climate geoengineering change public event mitigation dialogue decision talk member	Geoengineering (2010) SciHorizons Deliberative Panel (2006) Big Energy Shift (2008) Nanodialogues (2005) Small Talk (2005) Forensic Use of DNA (2007)
Class A4 (17.6%): The slippery slope to challenging our way of life	‘These technologies might bring some economic benefits and cheap food, but I don’t think we have the right to do this to the natural world. In the long term, I’m worried about whether there are safe, their effects on the environment and where this will lead’.	industrial biotechnology gm food crop environment product consumer release fuel	Industrial Biotechnology (2006) GM Foods (2002) BBSRC Synthetic Biology (2009)
Class A5 (17.8%): Where do we draw the moral line when we mess with humans?	‘The things that scientists can do with these technologies is not natural and I am not sure we have the moral right to do this. I can see that they might help some people, but I think I can only accept it if it will help humans with life-threatening conditions’.	animal human material embryo research create hybrid egg agree welfare	Animals Containing Human Material (2010) Hybrids and Chimera (2006) Stem Cell Dialogue (2007)

BBSRC: Biotechnology and Biological Sciences Research Council; GM: Genetically Modified.

#### Key features of the public discourses

##### (i) Groups of technologies, groups of views

The public discourses group around particular ‘types’ of technologies. Distinct discourses have emerged around drugs (Class A1); technologies with biomedical applications such as stem cells, nanoscience and synthetic biology (Class A2); non-biomedical technologies such as geoengineering, energy and non-medical nanoscience (Class A3); technologies that work with the genetic building blocks of life, such as synthetic biology and GM (Class A4); and those which involve combining human and animal material, such as hybrid embryos (Class A5).

Others have argued previously that people do not have attitudes to technology in general but have specific attitudes to particular technologies – for example, Evans and Durant (1995) discussed how people feel differently towards medical and other sciences Durant, Evans, and Thomas (1989); Bauer (2015) and Gaskell have described the difference in people’s attitudes towards ‘green’ and ‘red’ biotechnologies (Gaskell, 2001). Looking at public discussions of various technologies collectively, rather than being specific to each technology, attitudes appear to cluster in specific ways around broad ‘types’ of technologies. Such ‘clustering’ appears to be consistent with previous work describing how the public draw on their previous experience of other technologies to guide their views on new developments (Currall et al., 2006; Scheufele and Lewenstein, 2005; Slovic, 2010; Upham and Roberts, 2011). For example, people appeared to use nuclear and wind power as two ‘yardsticks’ to size up new environmental technologies (Lock et al., 2014).

Within these ‘clusters’, two factors appear to be particularly variable and characteristic – the role of nature and the shape of regulation:

(a) Role of nature
  The idea of nature and naturalness was important, particularly in helping participants decide to support a particular technology or not. Significantly, the public discourses appear to use three distinctly different concepts of nature or naturalness:

1. Ontological
  In discussions of gene technologies (especially those which mix the genes of different species), nature or naturalness is conceived as a state of being. Something is either natural or it is not. This distinction appears to be an important guide to what is and what is not desirable:
*It seems unsafe to carry out procedures which are unnatural, in the sense of being not possible by natural processes. (Hybrids and Chimera, 2006; A5)*
2. Ecological
  In relation to non-biomedical technologies such as geoengineering and agricultural biotechnology, nature is conceived in ecological terms – as a balanced system that we do not fully understand. Importantly, a view emerges that since we cannot anticipate the effect of human intervention on this system, it makes no sense to develop more technologies to address the previously unforeseen consequences of other technologies:
*Most participants believed that natural systems are balanced and self contained and that geoengineering should be considered in terms of how well it preserves natural systems. (Geoengineering dialogue, 2010; A3)*
  This ‘ecological’ conception of nature appears to draw upon the environmental movement’s narratives around ecosystems, Gaia and spiritual sense of ‘mother earth’ (Nordhaus and Shellenberger, 2007).
3. Deontological
  In discussions of less human-related aspects of genetic research, such as GM crops and synthetic biology, nature is talked about as a binary rule or law that we must obey or face consequences. This again appears to draw on environmentalist narratives around a ‘vengeful nature’ (Nordhaus and Shellenberger, 2007) and is often coupled with talk of nature as a system – upsetting the system will have consequences because you have broken the laws of nature:
*Some farmers in Pakistan went into debt to buy GM seeds, but they couldn’t sell the crops for enough money to afford the next year’s GM seed. GM technology violates natural law for economic gain. (FSA GM Foods, 2002; A4)*
  Others have identified a role for ‘nature’ or ‘naturalness’ in the public’s conceptions of technologies and risk. For example, Corner et al. (2012) found that a key factor driving public acceptance of different geoengineering procedures was the extent that a technology was perceived as supporting or imitating natural processes. Lock et al. (2014) found that a sense of ‘naturalness’ equated with a sense of ‘good’ when considering climate change technologies; Jasanoff (2005) describes how ‘modern’ and ‘unnatural’ became closely linked during the United Kingdom’s Bovine Spongiform Encephalopathy (BSE) crisis, with this connection later fuelling negative perceptions of GM; Wagner et al. (2001) found that expressions relating to ‘tampering with nature’ dominated focus group discussions about the dangers or hazards associated with GM, concluding that ‘the public debate on biotechnology is in fact deeply influenced by polarised views of nature’.
  Our findings echo these conclusions that a sense of nature is shaping views on technologies. Rather than being fixed value frames, however, we argue that these conceptions of nature act as models or schema which people can switch between depending on the technologies being discussed, helping people quickly form views of new technologies, the associated consequences and the way in which these should be governed or regulated.

(b) Regulation and governance

The way people talked about regulation also appeared to relate to the type of technology being discussed. Drugs (A1) were spoken of in terms of criminal law:

*Others argued that testing would be an invasion of privacy and that if the drugs were legal, there would be no need to conceal their use. (Drugsfutures, 2006; A1)*

For biomedical applications (A2), controlling and regulating the role of industry and the market were important:

*The biggest challenge to this area will be the tension between public and private investments as there is a move to commercialise academic research. (Stem Cell Dialogue, 2006; A2)*

Geoengineering and non-biomedical technologies (A3) were matters for government policy:

*Participants thought that government and other agencies should set a timetable for action. (Geoengineering, 2010; A3)*

Regulating GM (A4) was discussed both as being subject to the laws of nature, with consequences if they are not obeyed (as illustrated in quote above), and in terms of consumer relationships, with labelling an important word:

*They assumed at a certain point industrial biotechnology products would begin appearing in shops and that they would either be labelled, in which case they as consumers could make a choice about whether to buy them, or worse would not be labelled, thus forcing the consumer into unknown consumption of industrial biotechnology products. (Industrial Biotechnology, 2009; A5)*

Finally, regulating human genetic technologies (A5) was discussed in terms of morals – morality rather than legality should guide whether or not a technology is developed:

*The first [concern] was around the ethics of using foetal material for clinical or research purposes, with certain participants uncomfortable as to whether this was morally acceptable. (Stem Cells, 2007; A5)*

##### (ii) Social and ethical issues discussed as inherent to technologies

The way the public discourse cluster around technologies rather than ‘issues’ also indicates that the public discussed wider social and ethical issues in the context of the technologies themselves. Unlike in the expert and policy analysis (which we will discuss later), there was no class devoted to ‘aspirations’ or ‘risks’ or ‘explaining the science’, for example. Instead, aspirations and risks are found within the classes discussing particular ‘types’ of technologies. We argue that this suggests the public discourses consider the benefits and risks of technologies as fundamental parts of the technologies – different faces of the same coin, which cannot be separated.

##### (iii) Contingent optimism

Throughout, the public discourses conveyed a sense of progress and optimism about science, especially biomedical science, but coupled with a strong sense of contingency. How they felt about a particular science and technology depended upon how it was being used and the special circumstances of each case, rather than an inherent quality of the technology:

*Many appear to view a clear rational for the research as the key to determining whether it was acceptable or not. (Hybrids and Chimera, 2006; A5)*

The direction that the technology was taking society was also important in guiding discussions:

*In general, people were worried about the capacity for science to take things too far. (Industrial Biotechnology, 2006; A4)*

Biomedical applications of stem cells, synthetic biology and nanoscience were the most positively received, and in these cases, maximising the benefits and minimising the ‘problems’ associated with them is talked about as the priority. While risks are recognised, they are seen as worth taking if they lead to cures, particularly for life-threatening diseases:

*There was a view that there were inherent risks involved in developing new technologies but that if we were too careful with the development of nanotechnologies then this could lead to the field stagnating and loosing impetus. (Nanotechnology for Healthcare, 2008; A2)*

Other technologies (for instance, environmental technologies such as geoengineering) met with a stronger sense of scepticism or at least a greater need for balance:

*They asserted that one climate solution would not be enough to tackle climate change. The majority wanted to combine potentially several different international geoengineering approaches with international national and individual mitigation efforts. (Geoengineering, 2010; A3)*

Overall, the public discourses did not tend to form simple judgements of technologies, but instead to suggest that more information, consideration of different angles or perspectives and a balancing of different interests and needs were necessary.

##### (iv) Focus on people not technologies

People (rather than the technologies) were the focus throughout the public discourses. ‘Family’, ‘child’ and ‘parent’ were significant words when talking about drugs (A1); ‘patient’, ‘donor’ and ‘stakeholder’ were significant words in discussions around biomedicine (A2); ‘expert’, ‘policymaker’, ‘citizen’ and ‘scientist’ were significant words in discussions of geoengineering, energy and nanoscience (A3); ‘consumers’, ‘producers’ and ‘farmers’ were significant words in discussions of industrial biotechnology, GM and synthetic biology (A4); and ‘humans’ and ‘man’ were significant in discussing human embryology (A5).

##### (v) Role of industry accepted, but seen as a diverting force

Throughout the discourses, the role of industry and the private sector in science and innovation is acknowledged but treated with suspicion – as a corrupting influence that scientists may not be able to resist:

*Whist participants generally did not consider academic scientists as doing research with profit as the main motive, the potential allure of private sector investments and the relative inexperience of researchers in brokering effective business deals could mean that ideas and innovations get taken in directions that are much less socially beneficial. (BBSRC Synthetic Biology Dialogue, 2009; A2)*

*Participants were somewhat sceptical of the motivation of commercial inters and government to ensure that the fairest outcomes were reached. (Geoengineering dialogue, 2010; A3)*

This appears to tie in with discussions of science as being on a slippery slope and in some way out of control. Controlling or tempering this corrupting influence – keeping the focus of science onto the important problems – is spoken of as key role of government.

### B Expert discourses

The analysis of the 12 expert reports produced five classes, or five distinct discourses, summarised in Table 2.

**Table 2.** Summary of analysis of expert reports.

Class	Illustrative statement	Ten most significant words	Documents associated
Class B1 (17.16%) ‘Addressing public concerns on social and ethical issues’	‘Developments in this area bring with them a number of social and ethical issues which were identified by the public in our discussions and which must be addressed if we are to take full advantage of the opportunities offered by this technology’.	public issue nanotechnologies ethical dialogue science geoengineering scientific debate concern	Geoengineering (2009) Nanosciences (2004) UK DNA Database (2009) Nanodialogues Response (2007) Synthetic Biology Roadmap (2012)
Class B2 (21.44%): ‘GM reassurance’	‘GM crops will bring huge benefits to the UK. Most of the risks associated with them are either not based on the scientific evidence, are reversible or can be avoided. In fact, many of the possible problems are no worse than the problems associated with current practices anyway’.	crop gm plant herbicide gene flow breed resistance food variety	GM Science Review (2003)
Class B3 (24.60%) ‘Brain Science’	‘People already use psychoactive drugs – legally or illegal. New brain drugs like cognition enhancers will help treat mental illnesses and things like Alzheimer’s disease but the members of the public we talked to were concerned that they could be abused and lead to new problems’.	drug substance mental misuse treatment cognition person harm child disorder	Brain Science (2008)
Class B4 (17.61%) ‘The biomedical science bit’	‘Research in which human pluripotent stem cells are introduced into animal embryos will clarify the potential of such introduced cells to contribute to addressing questions around the advancement of knowledge into cancer and Parkinson’s’.	cell human embryo stem animal mouse tissue hybrid create embryonic	Hybrids and Chimera (2007) Animals Containing Human Material (2011) Stem Cells (2008)
Class B5 (20.1%), ‘Growth, economy and planet’	‘Investing in the right aspects of these technologies will allow UK to be competitive in the global market place, grow our economy and help us solve some serious problems ahead, like identifying new sources of energy’.	chemical nanoparticles manufacture nanotubes device industry production ib particle synthetic	Nanosciences (2004) Industrial Biotechnology (2005) Synthetic Biology (2009) Synthetic Biology Roadmap (2012)

GM: Genetically Modified; IB: Industrial Biotechnology.

#### Key features of expert discourses

##### (i) Positive discourses

Overall, the expert discourses were positive, discussing the potential of science to tackle the big problems ahead and to generate wealth:

*The ultimate goal of synthetic biology is to develop commercial applications that will benefit society. i.e. to design and build engineered biological systems that process information, manipulate chemicals, fabricate materials and structures that generate energy. (Synthetic Biology, 2009; B5)*

Where concerns exist about the technologies, these are seen as issues raised by the public, which can be addressed:

*If geoengineering is to play a role in reducing climate change an active and international programme of public and civil society dialogue will be required to identify and address concerns about potential environmental social and economic impacts and unintended consequences. (Geoengineering, 2009; B1)*

In the case of GM in particular (B2), the expert discourse is focused upon providing reassurance that this is a technology to be supported:

*Most of the possible negative impacts of GM crops on biodiversity are likely to be reversible. (GM Science review, 2003; B2)*

##### (ii) Separation of social and ethical issues

In contrast to the public discussions, the expert discussions treat social and ethical issues as epiphenomena – separate from the technologies themselves. This is evident in two ways.

First, in the CATA analysis, social and ethical issues were found in a distinct class (B1), rather than integrated into discussions of particular technologies.

Second, the text of the expert reports is also explicit that social and ethical issues are seen very much as epiphenomena that can be addressed or solved with more research, as the quotes below and above (relating to geoengineering) illustrate:

*The development of synthetic biology brings with it a key number of ethical and societal implications which must be identified and addressed. (Synthetic Biology, 2009; B1)*

##### (iii) Managing risk, closing debate and reassuring

Alongside treating social and ethical issues as epiphenomena, the expert discourses give a sense that risk can be managed and uncertainty turned into probability. The implication is that it is possible to address the issues raised by the public and move on with the science:

*Field studies may often be essential to quantify ecological exposure to hazards and thus estimate risk. (GM Science Review, 2003; B5)*

In addition to a discourse entirely devoted to providing reassurance on GM (B2), the discourse on social and ethical issues (B1) contains reassurance words such as ‘address’, ‘confidence’ and ‘encourage’.

The overarching tone of the discourses expressed in the expert reports is one of providing promise and reassurance that the benefits can be achieved with minimal risk or concern.

##### (iv) Regulation is described not prescribed

Regulation words in the expert discourses describe what regulation there is rather than what ought to be and to demonstrate that they are sufficient:

*Although the creation of true hybrids is permitted in the UK, it is illegal to keep or use hybrid embryos in vitro beyond very early developmental stages. (Animals Containing Human Material, 2011; B4)*

##### (v) Industry and economics important

A whole class/discourse is devoted to the role of science in industry and markets. Industry is discussed as not just a beneficiary of these new science and technologies but a key reason for pursuing them. This is often framed in a national context – ensuring the United Kingdom maintains a competitive edge over other countries. While the public discourse mentioned fairness as being important, the expert discourse gave no such indication, implying that these economic (and other) benefits are shared by everyone:

*In years to come, the UK’s success will increasingly be defined by our competitive edge in this and other knowledge intensive industries. (Industrial Biotechnology, 2005)*

##### (vi) Technical language and science focus

Technical language is used throughout the expert discourses, and the focus (except in class B1) is on the science itself:

*Cell replacement was by transplanting foetal ventral mesencephalic tissue. (Stem Cells, 2008; B4)*

### C Policy discourses

The analysis of the 11 texts making up Corpus C (Policy documents) produced four classes or discourses, summarised in Table 3.

**Table 3.** Summary of analysis of policy reports.

Class	Illustrative statement	Ten most significant words	Documents associated
Class C1 (16.72%) ‘Safety and choice so that we can get huge benefits from GM’	‘GM crops could offer real benefits to consumers and farmers in the future and our comprehensive research has found no reason to think they pose any risks to human health, nor are any less safe than conventional crops. We do need to monitor this for unforeseen problems though and consider each on a case-by-case basis but much will depend on whether consumers choose gm foods and on the ability of the regulatory system to continue to manage any risks effectively’.	gm crop herbicide conventional grow gene maize plant farmer acre	Government’s response to GM Nation Debate Defra’s Frequently Asked Questions
Class C2 (29.15%): Regulation of human embryology research	‘The government should open the door to research using human animal chimera or hybrid embryos, as it is likely to bring significant health benefits in the future. There is little opposition, besides that based on opposition to research on human embryos in general. Legal advice is needed to consider the humanness of embryos, so that it is clear whether such matters should be regulated by HFEA or another agency, and the regulation needs to provide a clear framework within which research can take place’.	embryo human hybrid chimera animal creation hfea act draft cytoplasmic	UK government’s response to the Joint Committee Report of the Human Tissues and Embryos Draft Bill (2007) House of Commons Select Committee Report on government proposals for the regulation of Hybrid and Chimera Embryos (2006)
Class C3 (29.08%): Anticipating and managing risks and adapting regulation, to ensure UK maintains an international lead	‘The government will ensure a coordinated approach to developing this technology, which will be reviewed at 5 and 10 year intervals. This approach will bring together a wide range of stakeholders and the public, so that we can anticipate, understand and manage potential risks, address public concerns and ensure the responsible development of these fields while maintaining our international competitiveness’.	commission nanotechnologies information member royal public section society system regulatory	Government responses to reports on nanoscience, geoengineering and regenerative medicine.
Class C4 (25.05%): Supporting technology transfer	‘The government recognises the importance of the UK’s science base in providing the new ideas and innovations for translation into applications. The government will provide funding for such research over the next decade and develop a strategy to support businesses in exploiting this’.	ib innovation council fund igt sector business pound industry bbsrc	UK government’s responses to reports on industrial biotechnology, the UK stem cell initiative, nanosciences and synthetic biology.

GM: Genetically Modified; HFEA: Human Fertilisation and Embryology Authority; IB: Industrial Biotechnology; BBSRC: Biotechnology and Biological Sciences Research Council.

#### Key features of policy discourses

##### (i) Focus on supporting science for economic benefits and light-touch governance

Overall, the focus of the policy discourses is on enabling science and innovation to grow the economy and maintain national competitiveness. The government’s roles are regulating and supporting the various areas of science, balancing interests, providing infrastructure and coordination, and ensuring no harm is done and not getting in the way. The discourses describe a governance, rather than an active government approach:

*Government and industry can work to create an encouraging and enabling political and economic framework to catalyse the growth of the market of IB produced products, processes and technologies. (Government Response to Industrial Biotechnology report, 2009; C4)*

##### (ii) Benefits of science assumed, not specified

Beyond economic benefits, the benefits of science were not specified. Applications are implied rather than described. Science is seen as an automatic and unquestionable producer of goods:

*Synthetic Biology could produce solutions to many of humanity’s most pressing issues and at the same time presents significant growth opportunities. (UK Government response on Synthetic Biology, 2012; C4)*

##### (iii) Uncertainty, risk and unforeseen consequences acknowledged but seen as manageable

The policy discourses acknowledge the possibility of uncertainty and unforeseen consequences, describing the need to monitor and look out for these and to adapt the regulation to cope with these new consequences. But, as in the expert discourses, risk and uncertainty are seen as a knowable, quantifiable and manageable, with the solution being more research:

*The Government shares the Royal Commission’s understanding that there is no evidence of actual harm resulting from the use of nanotechnologies, but accepts that this is a possibility and that there is a need to develop our understanding further. (UK Government response to RCEP report on nanoscience, 2009; C3)*

##### (iv) Social and ethical issues seen as separate and to be addressed by experts

Connected to the previous point, throughout the policy discourses social and ethical issues are acknowledged but, as with the expert discourse, they are treated as matters that need to be addressed in order for science to proceed and as epiphenomena that can be dealt with separately from the science itself:

*Science and society dialogue that will seek to ensure that we have a regulatory system which will address public concerns and which allows the development of nanotechnologies in a responsible and innovative way. (UK Government response to RCEP report on nanoscience, 2009; C3)*

While the concerns are seen as public matters, addressing them is seen as an expert matter or risk or law and expert sources are cited throughout the policy discourses. For example, C2, which discusses the legislation around animal–human hybrid embryos, recognises that whether hybrid embryos are animal or human is an important question. But this is seen as a legal matter to clarify who is responsible for regulating this research – if they are human embryos, then the Human Fertilisation and Embryology Authority (HFEA) is responsible; if animal, then the Home Office. In this way, the law is turned to in order to settle this very moral matter:

*HFEA receives revisited legal opinion on whether cytoplasmic hybrid embryos should be regarded as human for the purposes of the HFW Act and whether such creations would be prohibited or licensable under the act. (House of Commons Report on Hybrid and Chimera Embryos, 2006; C2)*

##### (v) Public dialogue mentioned but problematised

Public dialogue events are mentioned in the policy discourses, especially as ways of identifying public issues to be addressed. They are, however, problematised, with questions raised about their representativeness and calls for similar views to be sought from ‘experts’ in order to verify their validity:

*We have seen no conclusive evidence to indicate the true state of public opinion on the creation of animal human chimera. (House of Commons report on Hybrid and Chimera Embryos, 2006; C2)*

## 4. From discourses to sociotechnical imaginaries

Drawing back from the individual discourses described above, we explored and compared the key themes underpinning how the different actor groups understood science and technology and its place in our world, to build a picture of the underlying sociotechnical imaginaries. This is summarised in Table 4.

**Table 4.** Comparison of key features of public, expert and policy discourses.

	Public	Expert	Policy
Sense of progress	Positive sense of contingent progress	Positive enthusiasm	Positive enthusiasm – benefits assumed not specified
Focus	Focus on people; nature important	Focus on science and uses technical language	Focus on economies and markets, science and citizens
View of social and ethical issues	Social and ethical issues inherent parts of science and technologies	Social and ethical issues seen as epiphenomena	Social and ethical issues seen as epiphenomena
Risk and certainty	Technologies seen as uncertain, unpredictable and contingent	Technologies seen as predictable and manageable with enough research	Unpredictability and contingency of technologies acknowledged, but focus on management
Role of industry	Industry seen as a diverting force	Industry seen as a beneficiary of science	Industry seen as a funder and beneficiary of science
Role of government	Role of government in managing balance and regulating role of industry	Role of government described but not prescribed	Role of government in funding basic research and enabling private sector

We argue that this comparison indicates two different sociotechnical imaginaries: (1) an elite imaginary of ‘Science to the Rescue’, underpinning the policy and expert discourses, which (unquestioningly) sees science as solver of problems and deliverer of social goods, as a driver of the economy and of international competitiveness, social and ethical issues as matters of risk and understanding that can be quantified and managed with more research and which stand aside from the technologies themselves; and (2) a public ‘counter-imaginary’ of ‘contingent progress’ which sees science and technology as not only producing goods and solutions but also producing problems, where these benefits and downsides are inextricably bound to the technologies, industry is a necessary but distorting influence that needs to be managed, and a sense that science can get out of hand, compromise nature or challenge what it is to be human.

While there are distinct similarities between these two imaginaries, we argue that the differences are significant – particularly when thinking about the impact of public dialogue on policy.

First, this close alignment of expert and policy imaginaries suggests that public perspectives have had very little impact on policy. It is not just that policymakers have listened to expert advice on the safety and potential of technologies but that they share their core understanding of the place of science and technology in our lives.

Others have described this situation in different terms – for example, a lack of reflexivity in policymaking institutions and the predominance of technocratic viewpoints within them (Emery et al., 2014; Macnaghten and Chilvers, 2014; Welsh and Wynne, 2013) leading to such institutions seeing dialogue as an opportunity to gain trust for a predetermined approach, rather than to rethink their policies and practices (Chilvers, 2012; Macnaghten and Chilvers, 2014; Stirling, 2007; Thorpe and Gregory, 2010; Wynne, 2006). The tension between the sociotechnical imaginaries described here helps make sense and raises new perspectives on these conclusions. Perhaps, the public’s ambivalence or understanding of the contingency of science is seen as resistance or views that need to be ‘brought around’, as Thorpe and Gregory (2010) have argued. It is also possible that policymakers cannot accommodate such complex public perspectives if, as Hurlbut (2015) argues, the shape of regulatory structures is based upon scientific imaginaries of science, in which social and ethical issues are epiphenomena.

Beyond the underlying sociotechnical imaginaries, the rhetoric and ontology of the public discourses also offer possible explanations why they might not have been fully accounted for in policymaking. Hilgartner (2000) has reported how expert use of technical language is a way of displaying competence and credibility and therefore winning the confidence of audiences. Cook et al. (2004) described how the public can be seen to be making emotional (rather than rational) assessments of technologies and to, therefore, be vulnerable to manipulation by the press, non-governmental organisations (NGOs) and politicians. If these views are shared by policymakers, then the public’s focus on people and nature versus the technical focus of the expert discussions might work to undermine the credibility of the public reports as a source of advice.

## 5. Conclusion

Our analysis suggests that the United Kingdom’s public dialogue events over the last 10 years provide a rich source of insight into how the public talk about and come to know new science and technologies.

By looking at the content of the reports of public dialogue, we have identified a series of public discourses around new and emerging science and technologies. In particular, we found that public discourses cluster around particular groups of technologies and consider the risks and benefits of technologies as integral to the technologies themselves.

Comparing these public discourses with the scientific expert and policy discourses on the same topics, we found that the scientific expert discourses strongly overlap with the policy discourses, revealing an underlying ‘elite’ sociotechnical imaginary that we have described as ‘Science to the Rescue’. In this imaginary, science is a driver of our economy and competitiveness and can solve our problems and deliver social goods; social and ethical issues are public matters relating to risk and understanding that stand aside from the technologies themselves and can be quantified and resolved my more research or information.

In contrast, the public discourses are underpinned by a ‘counter-imaginary’ of ‘Contingent Progress’ in which science and technology are seen not only as producing goods and solutions but also as producing (unforeseen) problems, problems which are as inherent to the technologies as the benefits they bring, where industry is a necessary but distorting influence that needs to be managed by the state.

Rather than looking for direct lines between public dialogue and policy documents – notoriously problematic as there is often a long time-lag between a dialogue and policy being made, and because policy is subject to so many influences (Emery et al., 2014) – the sociotechnical imaginaries described in this article offer a useful framework for evaluating the impact of dialogue by considering whether the high level ideas and visions are reflected in policy. Indeed, we argue that these two distinct imaginaries demonstrate that public dialogue has had little impact on UK public policy.

In terms of understanding how these elite imaginaries have been agreed and come to dominance, as well as how they act to resist other perspectives, we have put forward some tentative mechanisms – that the machinery of policymaking has been built around this elite imaginary, for example. This text-based approach does, however, appear to have limitations in explaining such mechanisms. More qualitative research and drawing on concepts from political science that focus on the importance of groups and associations in shaping policy (such as work on Policy Networks (Rhodes, 1997) and the Advocacy Coalition Framework (ACF; Sabatier and Jenkins-Smith, 1993; Sabatier and Weible, 2006)) are likely to offer further insight.

Finally, we argue that this broad characterisation of three sets of discourses – and the patterns of views within it – could form a valuable baseline for the future research and exploration in this area and provide a useful framework into which the future case studies could be compared. How do these discourses compare to similar expert or policy discourses? Do they change over time? Will new technologies create new patterns or slot into these existing discourses? How do they compare across nations?

## Supplementary Material

Online Appendix
