# Supplementary material for: Science to the rescue or contingent progress? Comparing 10 years of public, expert and policy discourses on new and emerging science and technology in the United Kingdom
Source: Public Underst Sci. 2017 May 11;27(6):655–73. doi: 10.1177/0963662517706452 (PMC6055116; doi:10.1177/0963662517706452)
Supplement: Online Appendix [file online_appendix_14_feb_17.pdf]

Online Appendix

1. Documents included in the computer assisted text analysis

Corpus A: Public dialogue reports

Animals Containing Human Material (2010): Led by the Academy of Medical Sciences, this public dialogue formed part of a broader study on the scientific, social, ethical, safety and regulatory aspects of research involving non-human embryos and animals containing human material.

DrugsFutures (2006-2008): DrugsFutures was commissioned as part of the Academy of Medical Science's independent review of the societal, health, safety and environmental issues raised by scientific advances in brain science, addiction and drugs (BSAD).

Forensic Use of DNA (2007-2008): This project aimed to find out people's views on the forensic use of DNA, particularly in relation to the information held on the National DNA Database (NDNAD). Sponsored by the Human Genetics Commission (HGC).

Geoengineering (2010): The Natural Environment Research Council (NERC), in partnership with Sciencewise-ERC carried out this public dialogue to explore the views of the public in relation to geoengineering. In particular the moral, social and ethical implications of future research on geoengineering were assessed.

GM Foods (2002)*: This report presents The Food Standards Agency's contribution to the UK government's dialogue on GM foods, based on a citizen's jury. The dialogue set out to independently assess people's views, especially those whose voices are not often heard, on the acceptability of GM food and how this relates to consumer choice.

Hybrids and Chimeras (2006): This project was part of the Human Fertilisation and Embryology Authority's (HFEA) public and stakeholder consultation on hybrids and chimera embryos. This was carried out to guide the Authority's recommendations to Government on whether this type of research should be allowed in the future.

Nanodialogues (2005-2007): Nanodialogues was developed as a result of recommendations by the Royal Society and the Royal Academy of Engineering on how the Government should take forward the new and challenging area of nanotechnology research.

Nanojury (2003)*: The NanoJury brought together 20 randomly-chosen people from different backgrounds who will hear evidence about the role that nanotechnologies might play in their future.

Nanotechnology for healthcare (2008)*; Sponsored by the Engineering and Physical Sciences Research Council, this dialogue set out to take account of a wide set of societal views and inform nanotechnology research trajectories for healthcare

Public perceptions of industrial biotechnology (2006): IB is the application of bioscience (including genetically modified organisms) for the processing and production of chemicals, materials and energy. The aim of the project, sponsored by the Department for Business, Enterprise & Regulatory Reform (BERR), was to assess and understand the public perception and values around IB.

ScienceHorizons (2006-2007): Sciencehorizons was the public-facing part of a stakeholder consultation on the Wider Implications of Science and Technology (WIST) carried out by the Government Office for Science's Horizon Scanning Centre. Its aim was to explore the public's views on future applications of science and technology that emerged from two Horizon Scans which involved scientists and experts thinking about future science and technology.

SmallTalk (2005)*: SmallTalk was a series of public dialogue activities that explored the aspirations and concerns of the public and scientists around nanotechnologies.

Stem Cell dialogue (2007-2008): This project developed public dialogue activities around the science and social and ethical issues of stem cell research, with the aim of providing an understanding of views and concerns among diverse groups, including scientists, which could then feed into policy makers' strategic decision making.

Synthetic Biology (2009-2010): The Synthetic Biology Dialogue has been commissioned by BBSRC (Biotechnology and Biological Sciences Research Council) and EPSRC (Engineering and Physical Sciences Research Council), on behalf of RCUK, and will explore the public's views, concerns and aspirations around synthetic biology.

Synthetic Biology, Royal Academy of Engineering (2008)*: This report presents the findings of an exploratory public dialogue project, commissioned by the Royal Academy of Engineering (the Academy) and conducted by People Science and Policy Ltd (PSP) to explore uninformed and informed perceptions of and attitudes to synthetic biology in the UK.

The Big Energy Shift (2008-2009): The Big Energy Shift aimed to encourage people to discuss the way they insulate, heat and power their homes and communities. It took place in parallel with the formal consultations on the Heat and Energy Saving Strategy and Renewable Energy Strategy.

Corpus B: Expert reports

Animals Containing Human Materials (2011) Academy of Medical Sciences: The broader study on the scientific, social, ethical, safety and regulatory aspects of research involving non-human embryos and animals containing human material, into which the public dialogue fed.

Brain Science (2008) Academy of Medical Sciences: Report produced by a working group chaired by Prof Sir Gabriel Horn, to consider societal, health, safety and environmental issues raised by scientific advances in brain science, addiction and drugs (BSAD).

Geoengineering the climate: science, governance and uncertainty

GM Science Review First Report (2003) The GM Science Review Panel. An open review of the science relevant to GM crops and food based on interests and concerns of the public
Human Animal Hybrids (2007) HFEA: Final report of the HFEA review of human animal hybrids, which the public dialogue feeds into.

IB 2025 – Maximising UK Opportunities from Industrial Biotechnology in a Low Carbon Economy (May 2009). A report to Government by the industrial biotechnology innovation and growth team, which included members from academia and industry.

Nanodialogues reponse (2007): BBSRC and EPSRC joint response to the nanodialogues: engaging research councils

Nanosciences and nanotechnologies: Opportunities and Uncertainties (2004) Royal Society & Royal Academy of Engineering report looking at future of nanoscience. Included input from a public dialogue activity.

Nothing to Hide, Nothing to Fear: Balancing individual human rights and the public interest in governance of the National DNA Database (2009) Human Genetics Commission. Report which 'forensic use of DNA' dialogue fed into. Project

Royal Society (2009). Report into potential of geoengineering, which included element of public consultation.

Stem Cell Infrastructure Report (2008) The Medical Research Council (MRC). Considers the research landscape and national needs, to determine infrastructure support for the sector.

Synthetic Biology - scope, applications and implications (2009) Royal Academy of Engineering : Identifies the next steps to build on the UK's position in the field, create a regulatory framework and to explore, with the public, the ethical and societal issues involved. Includes outputs of the 2008 public dialogue.

Synthetic Biology Roadmap (2012) Produced by the UK Synthetic Biology Roadmap Coordination Group, which included a wide range of stakeholders, including academics, industrialists and, on behalf of the Research Councils, the Biotechnology and Biological Sciences Research Council (BBSRC), Engineering and Physical Sciences Research Council (EPSRC) and the Technology Strategy Board (TSB).

Corpus C: Policy reports

Genetically Modified Foods – Frequently Asked Questions (Defra 2004)

Government Response to the House of Commons, Science and Technology Committee 5th Report of Session 2009-10: The Regulation of Geoengineering

Government Response to the House of Lords Science and Technology Committee Inquiry into Regenerative Medicine (2013)

Government response to the Industrial Biotechnology – Innovation & Growth team report to Government (2009)

Government Response to the Report from the Joint Committee on the Human Tissue and Embryos (Draft) Bill (2007)

Government response to the UK Stem Cell Initiative report and recommendations (2007)

House of Commons Science and Technology Committee: Government proposals for the regulation of hybrid and chimera embryos. Fifth Report of Session 2006–07.

The GM Dialogue – Government Response (2004)

UK Government response to 'A Synthetic Biology Road Map for the UK' (Letter from Science Minister, 2012)

UK Government Response to The Royal Commission on Environmental Pollution (RCEP) Report "Novel Materials in the Environment: The Case Of Nanotechnology" (2009)

UK Government response to the Royal Society and Royal Academy of Engineering Report 'Nanoscience and nanotechnologies: opportunities and uncertainties' (2005)

2. 50 most significant words for each class produced by IRAMUTEQ analysis of public dialogue documents

Number of texts: 18
Number of text segments: 5592
Number of words: 9062
Number of occurrences: 205499
Average number of occurrences for each word: 22.677003
Number of lemmas: 6602
Number of active words: 6055
Number of supplementary words: 547
Number of classes 5
3999 segments classified of 5592 (71.51%)

**Class A1 15.68%**	**Class A2 25.48%**	**Class A3 23.38%**	**Class A4 17.65%**	**Class A5 17.80%**
drug	application	climate	industrial	animal
young	area	geoengineering	biotechnology	human
recreational	treatment	change	gm	material
outreach	biology	public	food	embryo
user	potential	event	crop	research
person	science	mitigation	environment	create
belfast	synthetic	dialogue	product	hybrid
illicit	disease	decision	consumer	egg
child	fund	talk	release	agree
parent	nanotechnology	member	fuel	welfare
education	therapy	activity	biofuels	respondent
addiction	field	scientist	natural	cytoplasmic
alcohol	private	report	bacterium	cell
harm	medical	information	land	disagree
enhancers	investment	engagement	eat	creation
family	patient	expert	stage	man
cognition	stem	scientific	impact	survey
school	device	policymakers	gmos	figure
legal	clinical	trust	production	tissue
crime	female	recommendation	enzyme	compare
heroin	significant	policy	concern	percent
workshop	control	explain	chemical	acceptable
teacher	council	panel	process	statement
exeter	cell	government	organism	brain
service	development	principle	unite	purpose
addict	innovation	opinion	oil	woman
cannabis	healthcare	engage	country	type
class	male	ask	unknown	ivf
vulnerable	vision	deployment	kingdom	suffer
cocaine	sector	issue	plant	consultation
nicotine	culture	technology	feedstocks	donate

age	commercial	session	fossil	genetic
liverpool	governance	communicate	farmer	experiment
glasgow	highlight	warm	produce	contain
addictive	profit	datum	barrier	nationally
classification	advance	discussion	environmental	reproductive
peer	stakeholder	science	producer	specie
project	instance	robotics	shelf	foetal
criminal	regulator	comment	labelling	half
prison	govern	organisation	reassurance	write
vulnerability	progress	shift	aid	amount
relay	cancer	independent	contamination	cow
feel	hope	jury	industry	cord
smoke	risk	maker	price	acceptability
substance	think	interest	traditional	difference
adhd	drive	attitude	manchester	organ
commit	nanotechnologies	evidence	bio	q1
kid	aspiration	credit	ecosystem	mitochondrion
line	technology	scale	landscape	somatic
drink	regard	clear	bioplastics	possibility

3. 50 most significant words for each class produced by IRAMUTEQ analysis of expert documents.

Number of texts: 12
Number of text segments: 14335
Number of words: 17791
Number of occurrences: 520547
Average number of occurrences of each word: 29.259007
Number of lemmas: 13889
Number of active words: 12876
Number of supplementary words: 1013
Number of classes: 5
9305 segments classified out of 14335 (64.91%)

**Class B1 (17.16%)**	**Class B2 (20.44%)**	**Class B3 (24.69%)**	**Class B4 (17.61%)**	**Class B5 (20.1%)**
public	crop	drug	cell	chemical
issue	gm	substance	human	nanoparticles
nanotechnologies	plant	mental	embryo	manufacture
ethical	herbicide	misuse	stem	nanotubes
dialogue	gene	treatment	animal	device
science	flow	cognition	mouse	industry
geoengineering	breed	person	tissue	production
scientific	resistance	harm	hybrid	ib
debate	food	child	create	particle
concern	variety	disorder	embryonic	synthetic
research	seed	enhancers	es	property
governance	ht	addiction	line	product
stakeholder	kingdom	participant	cytoplasmic	surface
workshop	unite	alcohol	egg	size
consultation	wild	brain	donor	material
society	weed	young	somatic	bio
technology	conventional	cognitive	nuclear	market
raise	pest	psychoactive	mitochondrial	industrial
uncertainty	wee	illness	oocyte	base
report	bt	recreational	chim	nanoscale
group	rape	al	creation	biology
question	biodiversity	user	mitochondrion	process
policy	insect	abuse	derive	large
work	oilseed	family	mtdna	chemistry
attitude	impact	individual	scnt	biological
council	farm	cocaine	research	sector
royal	relative	health	transplant	price
social	tolerant	illicit	reprogram	exposure
interest	toxin	age	germ	energy
panel	pollen	heroin	genome	computer

deliberative	maize	cannabis	pluripotent	measurement
commission	trait	school	vitro	company
review	grow	receptor	blastocyst	application
societal	field	smoke	ras	feedstocks
future	resistant	healthy	nucleus	engineer
decision	farmer	depression	immune	molecule
international	agricultural	prevalence	hesc	chip
meet	potato	psychological	ips	cosmetic
member	fee	intervention	cord	fuel
technological	habitat	death	blood	renewable
inform	soil	behavioural	sperm	waste
address	agriculture	schizophrenia	transfer	scenario
discussion	feed	social	clone	carbon
highlight	glyphosate	nicotine	stage	uk
scientist	bird	risk	interspecies	technology
acceptability	pollination	illegal	type	optical
expert	specie	survey	derivation	synthesis
academy	tolerance	adhd	therapy	atom
independent	beet	dependence	chimera	mass
forward	invasive	effect	bone	design
citizen	farmland	neurotransmitter	differentiate	nanometre

4. 50 most significant words for each class produced by IRAMUTEQ analysis of policy documents.

Number of texts: 11
Number of text segments: 2589
Number of words: 5909
Number of occurrences: 94673
Average number of occurrences for each word: 16.021831
Number of lemmas: 4470
Number of active words: 3785
Number of supplementary words: 685
Number of classes: 4
1561 segments classified out of 2589 (60.29%)

Class C1 (16.72%)	Class C2 (29.15%)	Class C3 (29.08%)	Class C4 (25.05%)
gm	embryo	commission	ib
crop	human	nanotechnologies	innovation
herbicide	hybrid	information	council
conventional	chimera	member	fund
grow	animal	royal	igt
gene	creation	public	sector
maize	hfea	section	business
plant	act	society	pound
farmer	draft	system	industry
acre	cytoplasmic	regulatory	bbsrc
farm	bill	dialogue	strategy
food	legislation	operation	board
cultivation	hfe	paragraph	department
benefit	law	share	leadership
generation	licence	confidence	forum
resistant	purpose	government	skill
tolerant	prohibit	approach	epsrc
person	fertilisation	inform	lead
soil	allow	effort	synthetic
conclude	research	european	programme
evaluation	fall	medicine	biology
risk	embryology	website	support
wildlife	remit	safety	technology
rape	parliament	early	initiative
oilseed	proposal	ensure	energy
effect	woman	understand	market
country	tell	view	product
cost	create	way	strategic
narrow	permit	price	sustainable

variety<br>commercial<br>agriculture<br>pest<br>fses<br>offer<br>unit<br>organic<br>biodiversity<br>farmland<br>fse<br>ecology<br>deep<br>study<br>result<br>suggest<br>scale<br>resistance	white<br>license<br>regulation<br>day<br>propose<br>inter<br>implantation<br>viability<br>revise<br>mix<br>legal<br>inquiry<br>regulate<br>committee<br>authority<br>regard<br>gamete<br>power	saving<br>hospital<br>enhance<br>stage<br>nanotechnology<br>response<br>international<br>therapy<br>maker<br>work	investment<br>project<br>engineer<br>industrial<br>centre<br>chemical<br>capability<br>work<br>new<br>facility<br>bio<br>rural<br>change<br>affair<br>uk<br>opportunity<br>procurement<br>academic
